# *In Vivo* Applications of CRISPR-Based Genome Editing in the Retina

**DOI:** 10.3389/fcell.2018.00053

**Published:** 2018-05-14

**Authors:** Wenhan Yu, Zhijian Wu

**Affiliations:** Ocular Gene Therapy Core, National Eye Institute, National Institutes of Health, Bethesda, MD, United States

**Keywords:** CRISPR, genome editing, gene therapy, retinal degeneration, photoreceptors, AAV vector

## Abstract

The rapidly evolving CRISPR-based genome editing technology is bringing revolutionary changes to the entirety of the life sciences. In this mini-review, we summarize the recent progress of *in vivo* applications of CRISPR genome editing in retinal studies. Non-viral and viral vector mediated delivery have been developed for temporary or persistent expression of CRISPR components in retinal cells. Although in theory CRISPR-based genome editing can correct a large number of mutant genes responsible for a variety of inherited retinal disorders (IRDs), precise gene modification relies on homology-directed repair (HDR)–the efficiency of which is not currently high enough for meaningful benefit. Development of CRISPR-based treatment for retinal diseases thus far has been mainly focused on gene knock-out or gene deletion in which the highly efficient non-homologous end joining (NHEJ) repair pathway is involved. Therapeutic benefits have been achieved in a few rodent models of retinal diseases following CRISPR treatment. The *in vivo* applications of CRISPR have also facilitated studies of gene function in the retina. As off-target events and immune responses are still the major concerns, continuous development of safer CRISPR genome editing systems is prerequisite for its clinical applications.

## Introduction

**Figure 1:**
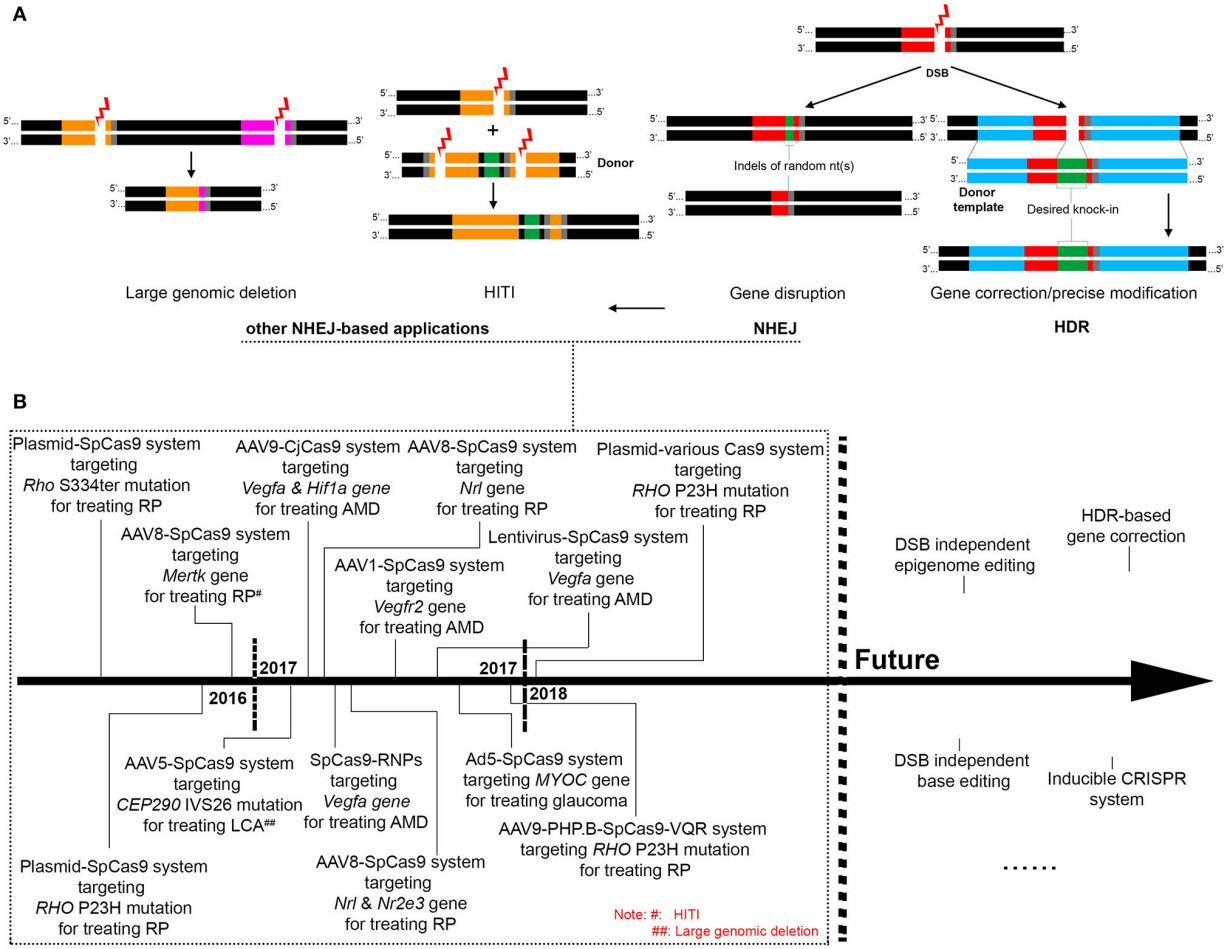
Overview of the mechanisms of CRISPR-based genome editing and its applications for treating retinal diseases. **(A)**. The mechanisms and patterns of CRISPR genome editing utilized in existing retinal studies, with black region indicating normal genomic DNA, gray region indicating the PAM motif, and red, orange and pink regions indicating different CRISPR-targeted sequences. **(B)**. Past and future applications of CRISPR genome editing for treating retinal diseases.

Genome editing is a group of techniques used to modify the genome of a cell or organism. It usually involves the introduction of an engineered nuclease leading to the generation of a double strand break (DSB) at a desired location on the genome, followed by an endogenous DNA repair process through non-homologous end joining (NHEJ) or homologous recombination (HR) (Figure 1A). NHEJ is a dominant DNA repair pathway that happens in all phases of the cell cycle. During NHEJ, insertion or deletion (indel) of random nucleotides can be introduced into the DSB site, which usually causes a reading frame-shift if the DSB is located in a coding sequence. Therefore, the error-prone NHEJ is often utilized to conduct gene disruption (or gene knock-out). In contrast, the error-free repair of DSBs by HR involves the copying of DNA from a homologous template, which is far less efficient and only occurs in late S phase or G2 phase of the cell cycle. Precise genome modification can be achieved by the HR pathway if a DNA template with homology arms is introduced to the cell together with the engineered nuclease, a process called homology-directed repair (HDR) (Thompson and Schild, 2001; Lieber, 2008, 2010; Figure 1A). Development of genome editing tools in recent years has been mainly focused on the exploration of novel engineered nucleases that are more easily accessible and enable more efficient and site-specific DSB generation. Thus, far four types of engineered nucleases, namely meganucleases, zinc-finger nucleases (ZFNs), transcription activator like effector nucleases (TALENs), and clustered regularly interspaced short palindromic repeats (CRISPR)/Cas nucleases, have been develop (Cox et al., 2015). Unlike the other genome editing tools that require engineered protein domains to target specific DNA sequences, the newly discovered CRISPR-Cas9 endonuclease is guided by an RNA of 20 nucleotides that recognizes the target DNA via Watson-Crick base paring. Because of the simplicity of its design, CRISPR/Cas9 has become the most popular genome editing tool and has been applied for a variety of research purposes including disease modeling (Dow, 2015; Tu et al., 2015), genetic screening (Sanjana et al., 2014; Hart et al., 2015), epigenome editing (Larson et al., 2013; Thakore et al., 2016), cell labeling (Chen et al., 2013; Ma et al., 2015; Nelles et al., 2016), and gene therapy (Yin et al., 2014; Long et al., 2016; Nelson et al., 2016; Tabebordbar et al., 2016).

The retina is the light-sensitive layer of tissue lining at the back of the eye and sends visual messages through the optic nerve to the brain. A variety of retinal disorders can cause irreversible blindness and visual impairment, affecting millions of people worldwide, with no effective treatment available. Inherited retinal disorders (IRDs) are caused by mutation (s) in one or more of over 200 different genes or loci (https://sph.uth.edu/retnet/sum-dis.htm), which could be treated by gene intervention including genome editing. Gene intervention could also modulate the disease pathways of multifactorial retinal diseases such as age-related macular degeneration (AMD), diabetic retinopathy, and glaucoma. Compared to the gene replacement (or gene augmentation) approach that is commonly adopted for treatment of recessive diseases, precise gene repair by genome editing is more attractive as it may permanently restore endogenous gene expression. Meanwhile, gene disruption by genome editing could be more efficient than the RNA interference–based approach for treatment of dominant diseases. However, *in vivo* genome editing in the retina was rarely conducted before the emergence of the CRISPR-based technology. This was not only because of the complexity in designing sequence-specific nucleases including meganucleases, ZFNs and TALENs, but also because of their large coding sequences hard to be delivered into retinal cells. In contrast, the Cas9 endonuclease gene, either alone, or together with the small guide RNA (sgRNA) expression cassette, is small enough to be packaged into one or two adeno-associated viral (AAV) vectors that are capable of transducing retinal cells efficiently. In this regard, *in vivo* application of CRISPR/Cas9 genome editing in the retina, though only at its infant stage, represents a significant step forward in developing new treatments for retinal diseases. In addition, for gene function studies, it provides an alternative to gene-knockout by the postnatal intervention of the genome. Although several reviews on similar topics have been published (Campbell and Hyde, 2017; Peddle and MacLaren, 2017; Peng et al., 2017), in this mini-review, we summarize the recent progress of the *in vivo* applications of CRISPR genome editing in the retina and focus our discussion on the *in vivo* delivery, applications in basic and pre-clinical studies, and challenges and future perspectives.

## Delivery of CRISPR components

Both viral and non-viral methods have been used for the delivery of CRISPR components to the retina. Electroporation to mice or rats at post-natal day 0 (P0) following subretinal injection of a plasmid encoding the CRISPR components was used in a few studies and efficient genome editing was detected in transfected retinal areas (Wang et al., 2014; Bakondi et al., 2016; Latella et al., 2016; Giannelli et al., 2017; Li et al., 2018). The transfected cell types may include photoreceptors, bipolar cells, Muller glial cells, and amacrine cells (Dhande and Crair, 2011). However, efficient transfection can only be achieved when electroporation is performed on newborn rodents in which retinal cells are in mitotic phase. Therefore, the electroporation approach may not be practical for therapeutic purposes, as retinal symptoms are usually present when retinal cells have already entered the postmitotic phase.

Thus far, the AAV vectors have been the most efficient tool for *in vivo* gene delivery to the retina and have been used in a few clinical studies of gene therapy for retinal diseases. AAV-mediated CRISPR delivery was firstly applied in mouse brains in 2015 (Swiech et al., 2015) and has since been widely used in a variety of tissues and organs including the retina. As the coding sequence of the most commonly used *Streptococcus pyogenes* Cas9 (SpCas9) approaches the packaging limit of an AAV vector, a dual vector system with one vector carrying the SpCas9 and the other carrying the single-guide RNA (sgRNA) is usually employed. A successful knockout of YFP gene in mouse retinal cells was reported using AAV type 2 (AAV2)-mediated CRISPR delivery (Hung et al., 2016). In a transgenic mouse line constitutively expressing YFP in inner retinal cells, an 84% reduction of YFP-positive cells was achieved in the eyes receiving treatment of CRISPR against YFP by intravitreal vector administration, compared to eyes receiving treatment of a control CRISPR. Our lab developed an AAV8-based CRISPR system for genome editing in mouse photoreceptors (Yu et al., 2017). Approximately 70% of the photoreceptors were transduced following subretinal administration, with gene disruption or indel formation detected in 43–98% of sgRNA-transduced cells (Yu et al., 2017). The availability of a large number of AAV variants would allow safer and more efficient CRISPR delivery to the retina. In a recent study, efficient genome editing in mouse photoreceptors was achieved following administration of a synthetic AAV9-PHP.B vector carrying CRISPR components via the less invasive intravitreal injection, due to the ability of retinal penetration of the new AAV capsid (Giannelli et al., 2017). In lieu of the dual AAV approach for CRISPR delivery, the shorter coding sequences of the newly discovered Cas9 nucleases from *Staphylococcus aureus* (SaCas9) (Ran et al., 2015) and *Campylobacter jejuni* (CjCas9) (Kim E et al., 2017) would allow the sgRNA cassette to be constructed into the same AAV vector. The one vector CRISPR approach is apparently more convenient in terms of vector production and could be more efficient in genome editing than the dual vector approach.

However, the AAV-mediated persistent expression of CRISPR components may be more likely to cause cytotoxicity, off-target events and/or immune responses, which are major concerns for *in vivo* applications of CRISPR. It is desirable that CRISPR components are no longer effective once they have fulfilled their task of generating a DSB. To this end, a self-limiting CRISPR/Cas9 system in which CRISPR recognition sites are incorporated into the SpCas9 expression cassette was developed, resulting in shortened duration of the Cas9 expression (Ruan et al., 2017). Another approach involved the use of cationic lipid-mediated delivery of CRISPR-Cas9 ribonucleoproteins (RNPs) consisting of a purified Cas9 protein and an sgRNA. Subretinal injection of RNPs to mice induced indels at the target site in retinal pigment epithelium (RPE) cells, with Cas9 protein completely degraded at day 3 post-injection (Kim K et al., 2017). Thus, transient expression of CRISPR components in the retina using the RNP approach may minimize the safety concerns. However, delivery of the RNPs to the neural retina did not seem to occur. For future development, efforts should be made toward expressing CRISPR components transiently in neural retinal cells such as photoreceptors in particular, as they are the primary cells affected in a large number of inherited and acquired retinal disorders.

## Therapeutic applications

In theory, precise gene modification relying on the HDR pathway can correct all gene defects responsible for IRDs. However, due to the postmitotic nature of most retinal cells, efficiency of HDR is too low to achieve a meaningful benefit. CRISPR-mediated gene disruption relying on the NHEJ pathway is the more commonly adopted approach in developing treatments for retinal diseases, especially those caused by dominant gene mutations (Figures 1A,B). In this regard, efficient discrimination between the mutant and the wide-type (WT) alleles, which ensures specific disruption of the mutant allele, is crucial to success. Mutations in the rhodopsin gene are one of the most frequent causes of IRDs, accounting for roughly 25% of autosomal dominant retinitis pigmentosa. Several studies have tested the use of CRISPR to knock out the mutant rhodopsin genes in rodent models. In a proof-of-concept study, electroporation of CRISPR components resulted in disruption of the murine S334ter allele in a transgenic rat model, which prevented retinal degeneration and improved visual function (Bakondi et al., 2016). However, the guide RNA designed in this study could not distinguish the murine WT allele from the mutant allele. In another study aiming at editing the most common mutation in human rhodopsin gene, CRISPR delivery by electroporation significantly reduced the amount of the mutant RHO protein in a transgenic mouse model carrying the human P23H mutant allele, although the sgRNAs were again not mutation-specific (Latella et al., 2016). Nevertheless, these studies demonstrate the potency of CRISPR-mediated *in vivo* knock-down of the mutant rhodopsin genes. In two most recent studies using CRISPR to edit the P23H rhodopsin mutation (Giannelli et al., 2017; Li et al., 2018), more practical approaches were developed toward future clinical applications. Mice with one WT allele and one P23H mutant allele were employed in both studies, mimicking patients with the dominant P23H mutation. After extensive testing of different Cas9 variants, Giannelli et al found that the use of an engineered SpCas9 (SpCas9 VQR) coupled with an sgRNA complementary to the P23H mutant but bearing a single mismatch to the WT allele resulted in efficient indel formation at only the mutant allele (Giannelli et al., 2017). Interestingly, in a different study, a significant level of indel formation at the WT allele was detected using identical SpCas9 VQR and sgRNA (Li et al., 2018). By employing a 5′ truncated 17 nucleotide sgRNA together with an improved version of SpCas9 (SpCas9 VRQR), efficient discrimination between the mutant and the WT allele was achieved. As the protospacer adjacent motif (PAM) sequence of the designed sgRNA was conserved between human and mouse genome, these two studies represent a step further toward human applications.

Mutations in the *CEP290* gene is one of the most common causes of Leber congenital amaurosis (LCA), a severe retinal degenerative disease with early onset and rapid progression. The most frequent mutation found in patients with *CEP290*-LCA is a deep intronic mutation (c.2991 + 1655A > G) in intron 26 of the *CEP290* gene (IVS26 mutation) that generates a cryptic splice donor site. A recent study showed that a pair of sgRNAs coupled with SpCas9 were highly efficient at removing the IVS26 mutation and restoring the expression of wild-type CEP290 in 293FT cells introduced with the mutation (Ruan et al., 2017). Effective deletion of an intronic fragment of the *Cep290* gene in the mouse retina was also achieved using AAV5-mediated delivery of CRISPR components, suggesting the *in vivo* therapeutic potential of the approach.

Instead of targeting specific pathogenic gene mutations, we tested a gene-independent approach for treatment of retinal degeneration by disrupting the neural retina leucine zipper (*Nrl*) gene encoding a transcription factor that specifies the rod cell fate. Following AAV8-mediated CRISPR delivery to the mouse retina, NRL expression was markedly reduced in rods, resulting in a gain of certain cone features and partial loss of rod function. The transduced rods presented with improved survival in the presence of mutations in rod-specific genes, consequently preventing secondary cone degeneration. Our results suggest that the CRISPR-mediated NRL disruption in rods could be developed into a viable treatment option for patients with retinal degenerative diseases (Yu et al., 2017). Similar results were obtained in a separate study in which both *Nrl* and *Nr2e3* genes were targeted (Zhu et al., 2017).

CRISPR-based genome editing could also be applied to treating multifactorial retinal diseases such as AMD, diabetic retinopathy, and glaucoma. Inhibition of neovascularization using anti- vascular endothelial growth factor (VEGF) agents has been an important treatment strategy for AMD and diabetic retinopathy, but current approaches require repetitive administration. To develop a long-term solution, two studies from a same group reported the disruption of the mouse *Vegfa* gene in RPE cells using either AAV9-mediated expression of CjCas9 or RNP-delivered SpCas9. Both CRISPR systems induced indels at a frequency of 20~30% in RPE cells, resulting in reduced area of laser-induced choroidal neovascularization (CNV) (Kim E et al., 2017; Kim K et al., 2017). Disruption of *Vegfa* gene was also achieved by lentiviral vector-mediated CRISPR delivery (Holmgaard et al., 2017). In another study, CRISPR-mediated depletion of VEGF receptor 2 (VEGFR2) in vascular endothelial cells (ECs) in the retina was explored. Intravitreal delivery of AAV1 vectors carrying an EC-specific promoter-driven SpCas9 gene and an sgRNA against *vegfr2* abrogated angiogenesis in the mouse models of oxygen-induced retinopathy and laser induced CNV (Huang et al., 2017). With regard to treatment of glaucoma, Jain et al. reported the use of an adenovirus-delivered CRISPR to knock down Myocilin (MYOC) with a gain-of-function mutation responsible for roughly 4% of primary open-angle glaucoma cases (Jain et al., 2017). In a transgenic mouse model carrying the human mutant *MYOC* gene, the treatment reduced the expression of the mutant protein in trabecular meshwork, resulting in alleviated endoplasmic reticulum stress, partial correction of the intraocular pressure phenotype and improved ganglion cell function.

A newly developed genome editing approach called homology-independent targeted integration (HITI) may broaden the scope of retinal diseases that could be treated by CRISPR. HITI relies on the NHEJ pathway to achieve gene knock-in at the desired genome location in both dividing and non-dividing cells (Figure 1A), with an efficiency much higher than the conventional HDR-mediated gene knock-in. In the Royal College of Surgeons rats in which the exon 2 of *Mertk* gene is deleted leading to retinal degeneration, the HITI approach allowed the successful insertion of exon 2 into intron 1, resulting in partial restoration of MERTK expression, retinal morphology and function (Suzuki et al., 2016). The existing applications of *in vivo* CRISPR genome editing in the retina are summarized in a chronological order in Figure 1B.

## Applications in gene function studies

Conditional gene knock-out is a technique used to eliminate a specific gene in a certain tissue in order to investigate gene function in live organisms including mice. Establishment of a mouse line for conditional knockout is time-consuming, as a pair of short sequences (e.g., loxP) recognizable by a specific recombinase (e.g., Cre) needs to be genetically engineered to flank the target gene. Compared to conditional gene knock-out, CRISPR-mediated post-natal *in vivo* gene disruption or deletion is more rapid and cost-effective, providing an alternative for gene function studies. To investigate the function of a 108 bp fragment (called B108) in the 5′ untranslated region of Blimp1, a transcription factor that regulates the rod vs. bipolar cell fate decision, Wang et al. applied CRISPR to delete the B108 fragment in retinal cells by electroporation to neonatal mice (Wang et al., 2014). Following treatment, more bipolar cells were observed in the transfected region, a result also seen in the *Blimp1* conditional knock-out retina, demonstrating the necessity of the B108 enhancer for proper Blimp1 regulation in the retina. In addition to investigations of individual genes, high-throughput analysis of gene function *in vivo* in the retina could be achieved in the future using CRISPR-based multiplex-mutagenesis (Sanchez-Rivera et al., 2014; Weber et al., 2015; Xu et al., 2017).

## Challenges and future perspectives

As the CRISPR components originated from prokaryotic cells, their expression may cause cytotoxicity and trigger immune responses in mammalians. Although persistent expression of CRISPR components did not cause obvious tissue damage in previous studies including our own (Platt et al., 2014; Yu et al., 2017), cellular and humoral immune responses in mice evoked by AAV-delivered Cas9 following intramuscular administration have indeed been observed (Chew et al., 2016). In a more recent study, presence of pre-existing adaptive immune responses to SaCas9 and/or SpCas9 was detected in humans (Charlesworth et al., in review), which may not only prevent the *in vivo* expression CRISPR components in humans following vector delivery, but also induce tissue or organ damage due to cytotoxic T cell response. However, CRISPR applications in the retina might be able to avoid the pre-existing immune responses if subretinal vector administration is used, as the subretinal space is segregated from the blood circulation. Nevertheless, cautions should be exercised, and more work needs to be done before CRISPR genome editing can be used *in vivo* in humans.

Another major concern associated with the CRISPR applications is the potential off-target mutagenesis. Although off-target events were not detected in most published *in vivo* applications of CRISPR, unbiased and highly sensitive whole-genome analysis methods should be used in future studies aimed at human clinical use. A few novel approaches have been developed to reduce off-target events. These include the use of truncated guide RNA molecules (Fu et al., 2014) and new variants or orthologues of Cas9 nucleases with higher fidelity such as eSpCas9 (Slaymaker et al., 2016), Cas9-HF (Kleinstiver et al., 2016), *Neisseria meningitidis* Cas9 (NmeCas9) (Lee et al., 2016), and CjCas9 (Kim E et al., 2017). Testing these approaches in the retina has just begun (Giannelli et al., 2017; Li et al., 2018).

The CRISPR genome editing technology is evolving at a rapid speed. The growing number of newly discovered or genetically engineered Cas9 nucleases that recognize different protospacer adjacent motif (PAM) sequences have greatly expanded the number of target sites available for genome editing (Komor et al., 2017). In addition to gene disruption/deletion and gene integration discussed above, the CRISPR-mediated epigenome editing is capable of regulating gene expression by modifying transcription and/or epigenetic state of specific DNA sequences without creating DSBs in the genome (Thakore et al., 2016; Liao et al., 2017). It usually involves the use of a fusion protein composed of a deactivated (or dead) Cas9 (dCas9) retaining the DNA binding function and an effector domain such as transcription enhancing factors, repressors, and epi- genetic modulators. With the delineation of epigenetic mechanisms involved in inherited and acquired retinal diseases, the CRISPR-mediated epigenome editing is anticipated to open a new avenue of treatment development for these diseases. To minimize immune responses and off-targeting events, inducible CRISPR systems in which the Cas nuclease or gRNA is controlled by a small molecule have been developed (Dow et al., 2015; de Solis et al., 2016) and can be applied in the retina. Meanwhile, efforts have been made to enhance the HDR efficiency for precise genome modification, though significant improvements have been limited to *in vitro* settings thus far (Maruyama et al., 2015). Another revolutionary technique derived from CRISPR is base editing which holds the potential to correct single nucleotide mutations without the need of DSB generation. The fusion of a cytidine deaminase such as APOBEC1 with a dCas9 enabled the conversion of cytosine (C) to thymine (T) at desired site with high specificity (Komor et al., 2016; Kim Y. B et al., 2017). The more recently developed adenine base editors (ABEs) allow efficient editing from A/T to C/G, which could be applied to correct almost half of all known human pathogenic SNPs (Gaudelli et al., 2017). *In vivo* base editing would be achieved if these editors can be modified to fit into AAV vectors. With these advancements, therapeutic CRISPR genome editing for retinal diseases will become a real treatment modality in the near future (Figure 1B).

## Author contributions

All authors listed have made a substantial, direct and intellectual contribution to the work, and approved it for publication.

### Conflict of interest statement

The authors declare that the research was conducted in the absence of any commercial or financial relationships that could be construed as a potential conflict of interest.
